# The effect of rabbit skeletal muscle on the large segment autologous bone ectopic preconstruction of vascularized bone flap

**DOI:** 10.3389/fbioe.2025.1639727

**Published:** 2025-10-28

**Authors:** Yanfeng Li, Mingwu Zhou, Qiyun Shi, Da Chen

**Affiliations:** ^1^ Luoyang Orthopedic-Traumatological Hospital of Henan Province (Henan Provincial Orthopedic Hospital), Zhengzhou, Henan, China; ^2^ Department of Orthopedic Surgery, Nanyang Central Hospital, Nanyang, Henan, China

**Keywords:** large-segment autologous bone, vascularized osteocutaneous flap, VEGF, BMP-2, ectopic prefabrication

## Abstract

**Aim of the study:**

To investigate the spatiotemporal secretion patterns of vascular endothelial growth factor (VEGF) and bone morphogenetic protein-2 (BMP-2) in rabbit skeletal muscle and their effects on the ectopic prefabrication of vascularized osteocutaneous flaps using large-segment autologous bone grafts.

**Methods:**

Seventy adult Chinese white rabbits (gender-neutral) were randomly assigned: 60 rabbits to the experimental group and 10 to the blank control group. In the experimental group, a 1.5 cm midshaft tibial segment from the left hindlimb was aseptically excised, stripped of soft tissues from the medullary cavity and bone surface, sterilized via high-temperature water bath, and implanted into the right rectus femoris muscle pouch for flap prefabrication. The control group underwent harvesting of a 1.5 cm left tibial midshaft segment and the entire right rectus femoris muscle without implantation. At 2, 4, 6, 8, 10, and 12 weeks postoperatively, 10 rabbits in the experimental group were euthanized to harvest the large-segment autologous bone and intact rectus femoris muscle. Specimens were analyzed for: 1. Macroscopic observation of bone remodeling; 2. Histological examination via hematoxylin-eosin (H&E) staining; 3. Immunofluorescence staining for CD34 (vascularization marker) and type I collagen (bone activity marker); 4. Western blotting to quantify VEGF and BMP-2 expression in muscle tissue. The control group was evaluated using identical methods.

**Results:**

1. Macroscopic Observation. The experimental group exhibited uneventful wound healing without inflammation. Fibroconnective tissues at the medullary cavity ends and bone cortex surface gradually thickened and adhered tightly over time. Upon dissection, increased number and depth of bone resorption lacunae and thinning of the bone cortex were observed. By week 8, the fibroconnective tissues wrapping the bone segment mimicked the thickness and tightness of the native periosteum, showing resistance to separation. 2. Histological Analysis (H&E Staining). Progressive bone tissue repair was observed in the experimental group: Week 2: Minimal fibrous granulation tissue on the bone surface and within the lumen. Week 4: Emergence of fibrous granulation tissue with sparse neovessels and cellular components. Week 6: Increased vascularity and cellularity, regular arrangement of bone vortices adjacent to blood vessels, appearance of mature osteocytes, expanded Haversian canals, and initiation of trabecular bone growth. From week 8 onward: Histological features matched fresh tibia, showing abundant vascular endothelial cells, osteocytes, and dense mature bone trabeculae. 3. Immunofluorescence Detection. CD34-positive blood vessels: The experimental group showed a “rise-then-slow-decline” trend, peaking at week 8 with significantly increased counts at each time point from week 2–8 compared to the prior week (P < 0.05). Compared to the experimental group at weeks 2, 4, 6, and 12, the baseline CD34-positive vessel number in the control group was significantly higher (P < 0.05). Type I collagen fluorescence intensity: An initial increase followed by a decline was observed in the experimental group, peaking at week 10 with significant enhancement from week 4–10 compared to the prior week (P < 0.05). Compared to the experimental group at weeks 2, 4, 6, and 12, the baseline Type I positive vessel number in the control group was significantly higher (P < 0.05). 4. Cytokine Secretion in Rectus Femoris Muscle. VEGF: Secretion in the experimental group followed an “increase-plateau-decrease” pattern, peaking at weeks 4–6 (significantly higher than weeks 2, 8, 10, and 12, P < 0.05) and exceeding control levels at weeks 2, 4, 6, and 8 (P < 0.05). BMP-2: Secretion peaked at week 6 (significantly higher than weeks 2, 8, 10, and 12, P < 0.05), being higher than the control group at weeks 2, 4, 6, and 8 (P < 0.05) but lower at weeks 10 and 12 (P < 0.05).

**Conclusion:**

1. During the ectopic prefabrication of vascularized osteocutaneous flaps using large-segment autologous bone, rabbit skeletal muscle secretes VEGF and BMP-2 in a spatiotemporally regulated manner. 2. VEGF and BMP-2 exhibit early-phase upregulation followed by gradual decline, playing critical roles in the prefabrication process. 3. Skeletal muscle promotes concurrent angiogenesis and enhanced bone activity during flap prefabrication. Completed vascularization at week 8 further supports sustained bone activity, facilitating functional bone regeneration.

## Highlights

• Dual Osteo-Angiogenic Capacity of Skeletal Muscle

First demonstration that rabbit skeletal muscle acts as an endogenous bioreactor, secreting vascular endothelial growth factor (VEGF) and bone morphogenetic protein-2 (BMP-2) to orchestrate both angiogenesis and osteogenesis during ectopic vascularized bone flap prefabrication.

• Novel Multimodal Evaluation System

Integrated histomorphometry (H&E staining), immunofluorescence quantification (CD34, type I collagen), and Western blotting for VEGF/BMP-2 to systematically assess spatiotemporal bone remodeling and cytokine dynamics.

• Clinical Translation Potential

Muscle-based prefabrication reduces traditional 6–12 months vascularization timelines, offering a promising strategy to recycle nonviable bone and address complex infected bone defects with minimized donor site morbidity.

## 1 Introduction

Infected bone defects, characterized by high morbidity, disability, and mortality rates, represent a major challenge in orthopedic surgery (Li et al., 2019; Ferrari et al., 2018). Conventional staged treatments are limited by prolonged duration, multiple surgeries, and suboptimal functional recovery (Yang et al., 2010; Luo et al., 2015), while cases involving critical-sized bone defects with soft tissue damage often face amputation risks (Xiao R., 2016; Yang et al., 2018). Although autologous osteocutaneous flap transplantation enables simultaneous reconstruction of composite defects, donor site morbidity restricts its clinical application (Li and Guo, 2008). Recently, extracorporeal sterilization and reimplantation of infected bone segments have shown considerable promise (Safak et al., 2000; Zhang, 2015). Moreover, ectopically vascularized prefabricated osteocutaneous flaps offer a novel strategy for recycling nonviable bone while overcoming anatomical limitations of conventional repair techniques (Zhang et al., 2016).

Skeletal muscle plays a dual role in bone regeneration: its anatomical structure provides an optimal microenvironment for ectopic bone maintenance, while its secretion of key cytokines (e.g., VEGF, BMP-2) regulates angiogenesis and osteogenesis (Fang et al., 2017; Lin and Wang, 2013). Indeed, muscle is increasingly recognized as an endocrine organ capable of secreting myokines (e.g., IGF-1, FGF-2, BMP-1) that positively influence bone formation, reinforcing its critical regulatory role in regeneration (Sui et al., 2024). Studies suggest that spatiotemporal coordination of VEGF and BMP-2 is critical for bone healing (Halawi and Morwood, 2015); however, the dynamic changes in myogenic factors during flap prefabrication and their regulatory mechanisms in autologous bone revascularization remain unclear. Angiogenesis and osteogenesis are now understood to be fundamentally interdependent during bone repair (Lv et al., 2023). Furthermore, recent bioengineering approaches using combined VEGF and BMP-2 delivery have achieved enhanced vascularized bone regeneration in critical defects (Orth et al., 2022), underscoring the importance of synchronizing angiogenic and osteogenic signals in healing.

Building on our previous success in establishing boiled bone and allograft-based prefabrication models (Yu, 2019), this study innovatively investigates the regulatory role of skeletal muscle in ectopic vascularization of large autologous bone segments. Using a rabbit autologous bone-muscle composite prefabrication model, we systematically evaluate: (1) temporal expression patterns of VEGF/BMP-2 in muscle tissue; (2) degree of vascularization and bone viability evolution; and (3) spatiotemporal correlation between myogenic factors and bone regeneration. To our knowledge, no prior study has comprehensively examined muscle-derived VEGF and BMP-2 dynamics during ectopic bone prefabrication *in vivo*, highlighting the novelty of our work in context (Kouba et al., 2022). The findings will provide a theoretical foundation for optimizing prefabricated osteocutaneous flap techniques, advancing personalized treatment strategies for infected bone defects.

## 2 Materials and methods

### 2.1 Experimental animals

Healthy white rabbits (n = 70) weighing 1.8–2.2 kg (mean weight = 2.0 ± 0.2 kg) were supplied by Animal Experiment Center of Henan University of Traditional Chinese Medicine, China (license No. 0007851). The animals were maintained at a mean temperature of 22 °C ± 1 °C, 50% ± 1% humidity, and a photoperiod of 12-h light/12-h dark cycle. All rabbits were performed in accordance with the guide for the Care and Use of Laboratory Animals of National Institutes of Health (Kastenmayer et al., 2012).

### 2.2 Key experimental reagents

#### 2.2.1 Basic reagents

4% (w/v) sodium pentobarbital (anesthetic, self-prepared), 0.5% povidone-iodine (Xinxiang Pharmaceutical Co.), 20% hydrogen peroxide (Shanghai Mengya Biotechnology).

#### 2.2.2 Animal treatment

Gentamicin (Beijing Kairuiqi Biotechnology, 80,000 U/2 mL), depilatory agent (Jinan Xiangtai Chemical Co.).

#### 2.2.3 Routine solutions

0.01 M PBS (pH 7.2–7.4, Shanghai Aiyan Biotechnology), 10% formalin (self-prepared from 40% formaldehyde), physiological saline (Shanghai Mengya Biotechnology).

#### 2.2.4 Molecular biology reagents

Lysis buffer, BCA protein assay kit, SDS-PAGE gel kit (Henan Cold spring Harbor Biotechnology); primary antibodies for VEGF and BMP-2 (Abcam, United States) and GAPDH (Wuhan Cloud-Clone Corp.); electrophoresis reagents including TEMED, ammonium persulfate (BBI Life Sciences), glycine (AMRESCO).

#### 2.2.5 Histological reagents

H&E staining kit (Beijing Kairuiqi Biotechnology); immunohistochemistry DAB kit and S-P9000 kit (Shanghai Yubo Biotechnology); decalcifying solution (10% EDTA), chloroform (Henan Hengsheng Chemical), ethanol gradients for dehydration.

### 2.3 Critical instruments and equipment

#### 2.3.1 Surgical operation system

6-hole metal bone plate (6 × 36 mm, Shanghai Sanwei Medical Devices); 1.0 mm Kirschner wire (Shenzhen Skada Metal Products); medical oscillating saw (Shanghai Huisheng Co.); electro-pneumatic drill (Zhengzhou Zhongshi Dichuang, YDJZ-II); digital subtraction angiography system (GE, United States); medical image analysis software (Beijing Jiayuan Xingye Technology).

#### 2.3.2 Sample processing

Paraffin microtome (Leica RM2235, Germany); high-speed centrifuge (Thermo Fisher MR23i, United States); benchtop refrigerated centrifuge; −80 °C and −20 °C freezers (NUAIRE, Japan; Shanghai Shiwei); thermostatic water bath (DHG-9031, Shanghai Fuzes).

#### 2.3.3 Molecular analysis

Vertical electrophoresis and transfer systems (ABI, United States); spectrophotometer (Beijing Delika Biotechnology); gel imaging analysis system (Shenzhen High-Speed Precision Instruments); laminar flow hood (SW-CJ-1FD, Guangzhou Haohan Instruments).

#### 2.3.4 Sterilization

High-pressure steam sterilizer (Shandong DTS Machinery); PVDF membranes (Wuhan Cloud-Clone); syringe filters (Shanghai Tusen).

### 2.4 Animal model preparation

#### 2.4.1 Preoperative preparation

Rabbits were acclimated in individual cages for 1 week before surgery. Gentamicin (10,000 U/kg) was administered intramuscularly 24 h before surgery for infection prophylaxis. Food and water were withheld overnight prior to anesthesia. On the day of surgery, anesthesia was induced via intraperitoneal injection of 3% sodium pentobarbital (30 mg/kg), with supplemental doses (10 mg/kg) administered as needed to maintain anesthesia. The surgical areas (left tibia and right thigh) were shaved, and limbs were immobilized in a supine position.

#### 2.4.2 Surgical procedure

In the experimental group (n = 60), after achieving adequate anesthesia and sterile preparation with povidone-iodine, a 4 cm incision was made along the medial midshaft of the left tibia. Blunt dissection was used to separate muscle and expose the midshaft. A 1.5 cm segment of the tibial midshaft (centered at the diaphysis) was resected using an oscillating saw. The bone defect in the left tibia was stabilized with a 6-hole straight metal bone plate (6 × 36 mm) fixed with Kirschner wires. The excised bone segment was stripped of periosteum and soft tissue, sterilized in a 60 °C water bath for 30 min (adapting an established prefabrication method), and then implanted into a muscle pouch created by bluntly separating the right rectus femoris muscle. The devitalized autologous bone segment was secured within the muscle with absorbable sutures. The incisions were closed in layers and dressed aseptically. This surgical design was based on prior heterotopic bone implantation models in rabbits, following the principle of vascular induction described by Safak et al. (2000).

In the blank control group (n = 10), after anesthesia, a 1.5 cm segment of left tibial midshaft was similarly resected. The animals were then euthanized by air embolism without muscle implantation. The harvested tibial segment was immediately fixed in 10% formalin (representing the 0-week baseline bone sample for histology), and the entire right rectus femoris muscle was excised and stored at −80 °C as the 0-week baseline muscle sample for biochemical analysis.

#### 2.4.3 Postoperative care

Rabbits were allowed to recover in individual cages with free access to food and water. Gentamicin (10,000 U/kg i. m., twice daily) was administered for 5 days post-surgery. Wound healing, appetite, and behavior were monitored daily. Three rabbits died of postoperative anorexia on day 2 and were excluded (replaced by new subjects to maintain group size). All remaining animals exhibited normal activity, good wound healing, and no signs of infection or distress.

#### 2.4.4 Observation timeline

At 2, 4, 6, 8, 10, and 12 weeks postoperatively, 10 rabbits per time point were humanely euthanized by overdose of 3% sodium pentobarbital (150 mg/kg, intravenous injection), in accordance with the guidelines approved by the Ethics Committee of Xinxiang Medical University and the National Institutes of Health Guide for the Care and Use of Laboratory Animals. The prefabricated autologous bone segments were retrieved through the original thigh incision for evaluation. Each retrieved bone segment (with its surrounding muscle and fibrous tissue) was examined macroscopically, then fixed in 10% formalin for 48 h. After fixation, samples were decalcified in 10% EDTA, processed, and paraffin-embedded. Sections (5 μm) were prepared for histological and immunofluorescence analyses. Concurrently, rectus femoris muscle tissue from the experimental site was collected for protein analysis. The control group baseline specimens (bone and muscle at 0 weeks) were processed identically for comparison.

### 2.5 Evaluation methods

#### 2.5.1 Macroscopic assessment

Retrieved bone segments were inspected for gross healing and remodeling changes. Key observations included the presence of inflammatory exudates, the extent of fibrous tissue encapsulation around the bone, and any visible cortical or medullary changes. Control group tibial segments preserved in formalin were examined for periosteal integrity and surface morphology as baseline references.

#### 2.5.2 Histological examination (H&E staining)

Formalin-fixed, decalcified bone samples were embedded in paraffin and sectioned at 5 μm. Sections were deparaffinized in xylene (I–III) and rehydrated through graded ethanol series. Hematoxylin staining was applied for 5 min, followed by eosin for 5 min. Sections were then dehydrated and coverslipped. Hematoxylin and eosin staining was performed according to standard protocols to ensure consistent visualization of tissue morphology (Fischer et al., 2008). Stained sections were evaluated under light microscopy for new bone formation, fibrous tissue presence, vascular structures, and cellular characteristics.

#### 2.5.3 Immunofluorescence staining

Paraffin sections were used for fluorescence immunohistochemistry targeting CD34 (a vascular endothelial marker) and type I collagen (an osteogenesis marker). After deparaffinization and rehydration, antigen retrieval was performed in 0.01 M citrate buffer (pH 6.0) at 95 °C for 10 min. Sections were cooled and then treated with 3% H_2_O_2_ for 20 min to quench endogenous peroxidase (for subsequent immunodetection steps). Non-specific binding was blocked with normal goat serum. Sections were incubated overnight at 4 °C with primary antibodies: mouse anti-CD34 (1:200) and rabbit anti-type I collagen (1:100) (both from Antibody Diagnostica Inc.). After washing in PBS, sections were incubated with appropriate fluorophore-conjugated secondary antibodies (Cy3-conjugated goat anti-mouse IgG and FITC-conjugated goat anti-rabbit IgG, respectively) at 37 °C for 2 h in the dark. Slides were counterstained with DAPI to visualize nuclei and mounted with an anti-fade medium. This immunofluorescence procedure follows an established paraffin-section protocol optimized to yield specific fluorescent signals with minimal background (Zaqout et al., 2020). Negative controls (omitting primary antibody) were included to verify specificity. Immunofluorescence images were captured using a confocal fluorescence microscope under consistent exposure settings. Quantitative analysis of fluorescence was conducted using image analysis software: CD34-positive microvessel density was manually counted in five random high-power fields (HPF, 100×) per sample, and mean values were calculated. For type I collagen, the average fluorescence intensity in five random fields was measured and normalized (arbitrary units).

#### 2.5.4 Western blot analysis

Rectus femoris muscle samples (approximately 100 mg) were snap-frozen in liquid nitrogen and stored at −80 °C until protein extraction. Frozen tissues were pulverized and homogenized in ice-cold lysis buffer (1:10 w/v) containing protease inhibitors. Homogenates were centrifuged at 12,000×g for 10 min at 4 °C. The supernatants were collected, and protein concentrations were determined by BCA assay using bovine serum albumin standards. Equal amounts of protein (30 μg per lane) were mixed with 2× Laemmli sample buffer (containing SDS and β-mercaptoethanol), boiled for 5 min, and loaded onto a 12% SDS-polyacrylamide gel. Electrophoresis was performed (initial 70 V, then 120 V) until the tracking dye reached the bottom. Proteins were then electrotransferred onto PVDF membranes (0.22 μm pore size) at 60 V for 2 h. Membranes were blocked with 5% non-fat milk in TBST (TBS +0.1% Tween-20) for 1 h at room temperature, then incubated overnight at 4 °C with primary antibodies against VEGF (1:1000), BMP-2 (1:1000), and GAPDH (1:5000) as a loading control. After washing, membranes were incubated with HRP-conjugated secondary antibodies (1:5000) for 1 h at room temperature. Signal detection was carried out using an enhanced chemiluminescence (ECL) substrate, and images were captured on an imaging system. Band intensities were quantified by densitometry; VEGF and BMP-2 levels were normalized to GAPDH. Western blotting was carried out following established techniques, ensuring equal protein loading and specific antibody detection of target bands (Burnett and e, 1981).

### 2.6 Statistical analysis

Data were analyzed with SPSS 23.0 (IBM Corp.). All quantitative results were expressed as mean ± standard deviation (x̄ ± s). A p-value <0.05 was considered statistically significant. For comparisons of the experimental group across multiple time points, one-way ANOVA followed by *post hoc* LSD tests were used. For comparisons between the experimental and control groups at each time point, independent-samples t-tests were applied. Repeated measures ANOVA was used where appropriate for time-course data. Significance indicators in figures are as follows: a indicates comparison between a given time point and the previous time point within the experimental group (p < 0.05); * indicates comparison between experimental and control groups at the same time point (p < 0.05).

## 3 Results

### 3.1 Gross specimen observations

**FIGURE 1:**
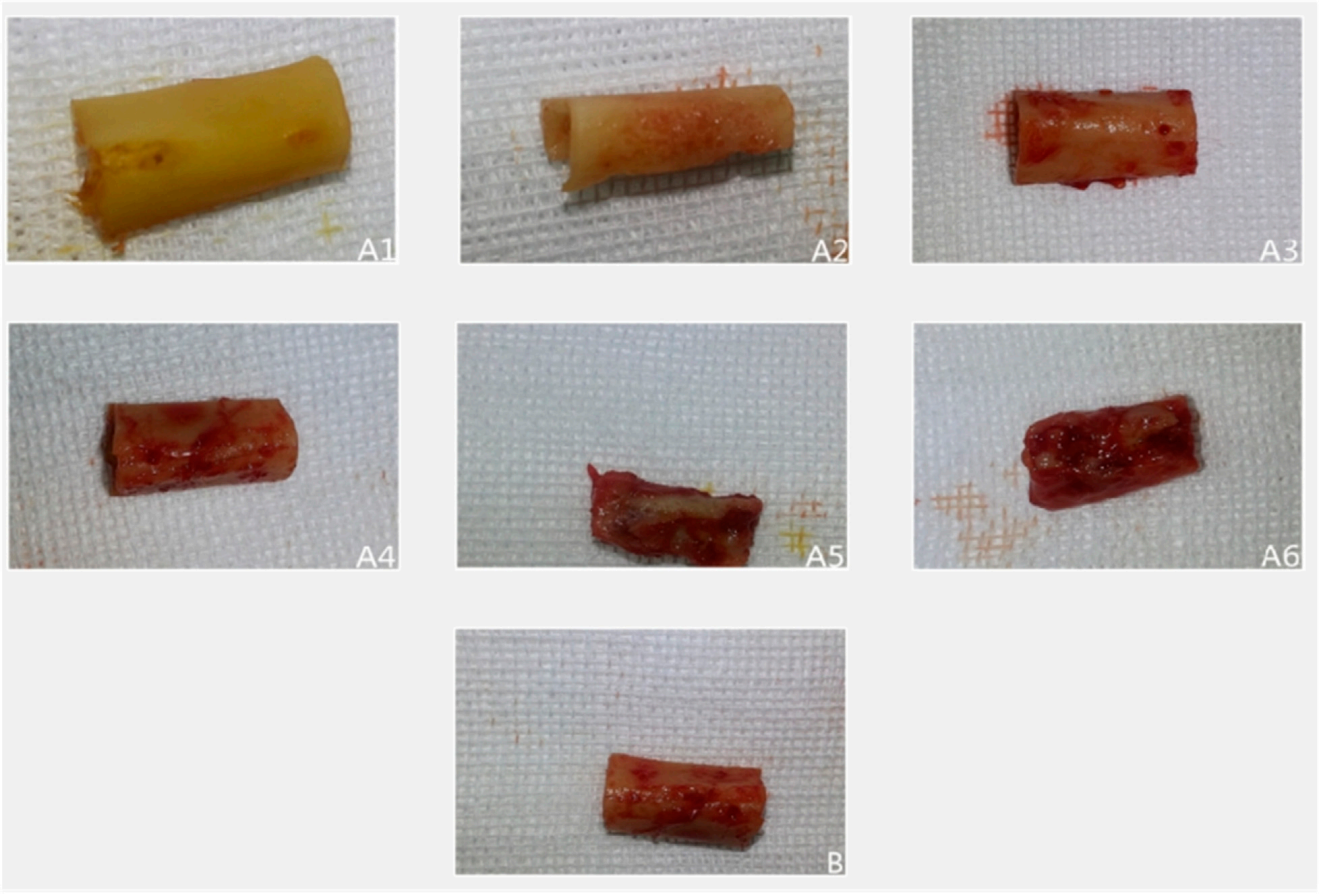
Illustrates representative gross specimens over time. In the experimental group (**(A1–A6)** corresponding to 2, 4, 6, 8, 10, 12 weeks), progressive fibrous encapsulation and cortical changes are evident. In the control group **(B)**, the baseline bone segment retains a smooth cortex without fibrous adhesion or resorption.

All surgical incisions in the experimental group healed uneventfully, with no signs of inflammation throughout the observation period (Figure 1). Temporal analysis revealed a progressive evolution of periosteal changes in the experimental group: At 2 weeks, no fibrous membrane encapsulation was detectable. By 4 weeks, a thin fibrous membrane had formed, which could be easily separated from the bone surface. Membrane thickness increased notably by 6 weeks, accompanied by minor soft tissue invasion into the medullary cavity; mild bone resorption pits became apparent upon membrane separation. At 8 weeks, the membrane exhibited significant thickening and strong adhesion to the underlying bone, with multiple resorption pits observed post-separation. These changes intensified by 10 weeks, characterized by further membrane thickening and increased density/depth of resorption pits. By 12 weeks, membrane thickness stabilized compared to Week 10, but adhesion strength increased substantially, concurrent with progressive cortical thinning and deeper resorption pits.

In the control group, bone segments developed periosteal encapsulation with thickness and adhesion strength comparable to Weeks 8–10 in the experimental group. However, distinctively, control specimens maintained smooth bone surfaces entirely devoid of resorption pits, suggesting an absence of active bone remodeling processes observed in the experimental cohort.

### 3.2 Histological analysis by HE staining

In the experimental group, temporal histological changes revealed a dynamic bone remodeling process. At Week 2, minimal fibrogranulation tissue was observed on bone surfaces. By Week 4, fibrogranulation tissue became evident, accompanied by small lacunae, sparse neovascularization, and scattered cellular components. Week 6 marked a pivotal transition: vascularity and cellularity increased markedly, while regularly arranged bone trabeculae containing mature osteocytes emerged alongside enlarged Haversian canals, signaling initial trabecular bone formation. By Week 8, Haversian canals further expanded, harboring abundant vascular cells, and well-organized bone lamellae were populated by numerous osteoclasts and osteoblasts, indicative of active remodeling. From Weeks 10–12, vascularity within Haversian canals decreased compared to Week 8, while canal diameters continued to enlarge, culminating in mature trabecular bone formation.

**FIGURE 2:**
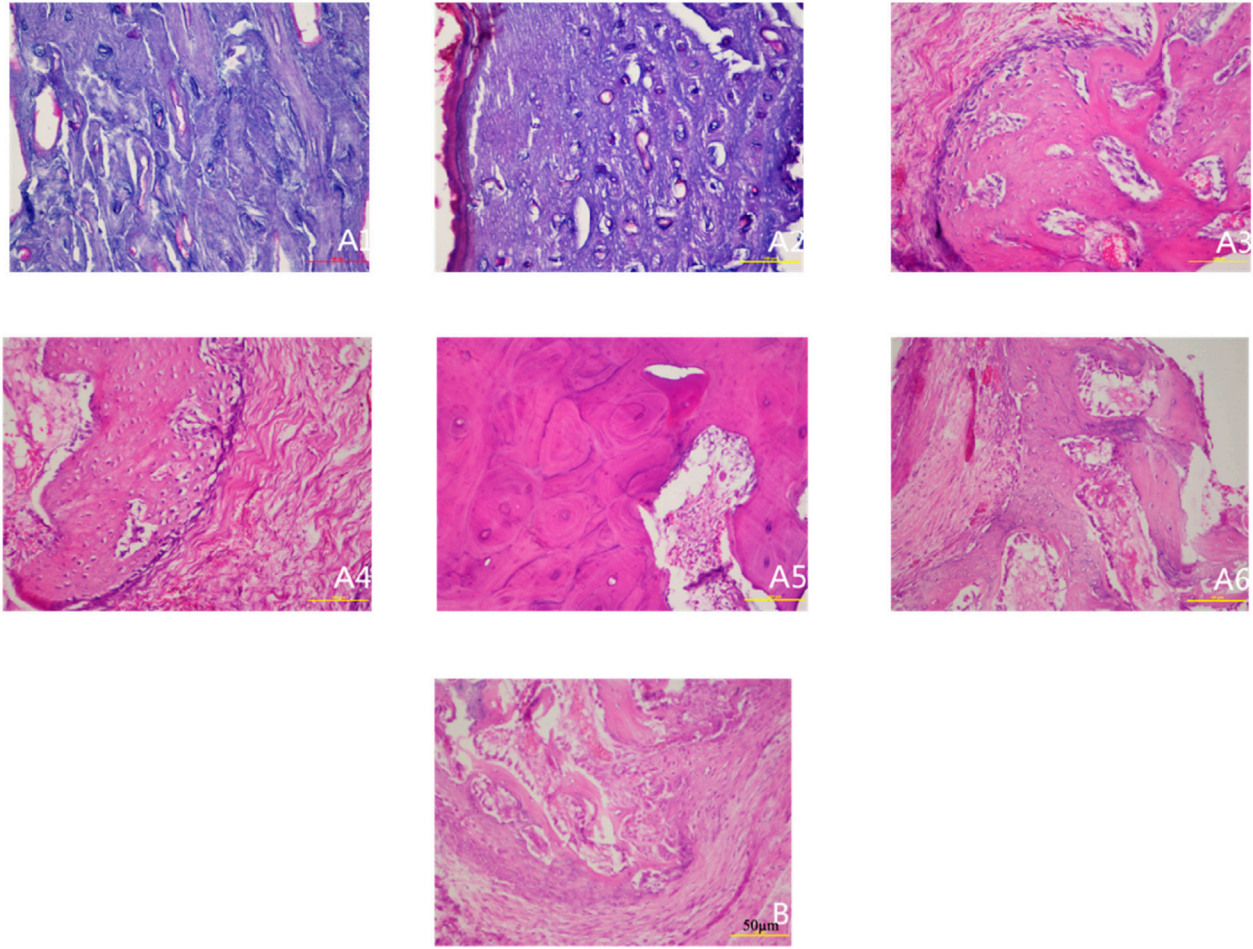
Histological observation of specimens at different postoperative time points (HE staining ×200). Note: **(A1–A6)** represent 2, 4, 6, 8, 10, and 12 weeks postoperatively in the experimental group; **(B)** represents the control group.

In contrast, control specimens (baseline tibia) exhibited a typical mature compact bone structure with organized Haversian systems and no evidence of injury or repair. The control periosteum contained normal vascular and cellular elements but showed no signs of the reactive changes or new bone formation observed in the experimental group. The overall histology of control bone remained in a stable, quiescent state, highlighting the lack of remodeling activity. (No representative control-group photomicrograph is shown in Figure 2; instead, the control group’s stable histological features are described textually above.)

### 3.3 Immunofluorescence analysis of CD34-Positive vasculature

**FIGURE 3:**
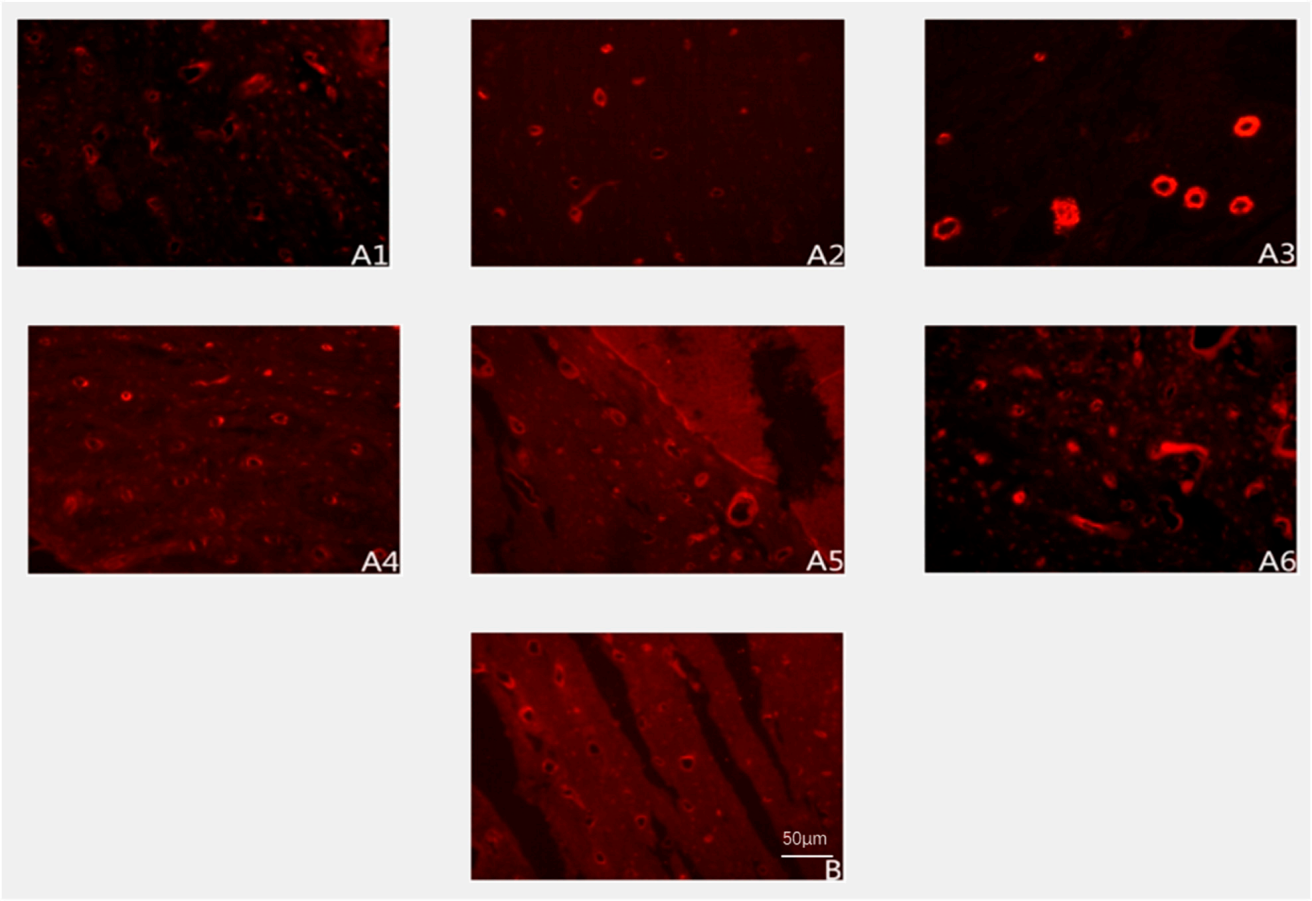
Expression of CD34-positive blood vessels at different postoperative time points (immunofluorescence staining ×200). Note: **(A1–A6)** represent 2, 4, 6, 8, 10, and 12 weeks postoperatively in the experimental group; **(B)** represents the control group.

The experimental group exhibited a distinct temporal pattern of angiogenesis, characterized by a progressive increase in CD34-positive vessel density peaking at week 8, followed by a gradual decline (Figure 3). Within-group comparisons revealed statistically significant increments in vascular density between consecutive time points from week 2 to week 8 (week 2 vs. 4: P < 0.05; week 4 vs. 6: P < 0.05; week 6 vs. 8: P < 0.05). However, no significant differences were observed between week 8 and 10 (P > 0.05) or week 10 and 12 (P > 0.05), indicating stabilization of vascular networks post-peak.

**FIGURE 4:**
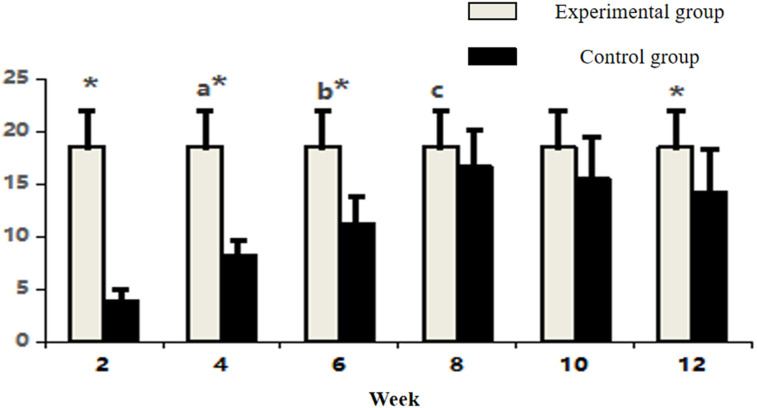
Comparative count of CD34-positive vasculature in osseous tissue across groups. Note: Within-group comparisons showed significant differences between 4-week and 2-week time points (a, P < 0.05), between 6-week and 4-week time points (b, P < 0.05), and between 8-week and 6-week time points (c, P < 0.05). Asterisk (*) indicates statistically significant differences (P < 0.05) between the Experimental and Control groups at corresponding time points.

Between-group comparisons demonstrated that control specimens maintained significantly higher vascularity than the experimental group at early and late phases (week 2, 4, 6, and 12: P < 0.05). Notably, vascular densities converged between groups at weeks 8 and 10 (P > 0.05) (Figure 4; Table 1), coinciding with the experimental group’s peak angiogenic activity. This temporal overlap suggests that the prefabrication process transiently achieves vascular parity with native bone architecture before subsequent regression.

**TABLE 1:** Comparative count of CD34-positive vasculature in osseous tissue across groups (n = 10,‾x ± s).

Time	Experimental group	Control group
2 Week	3.90 ± 1.02	18.50 ± 3.47*
4 Week	8.20 ± 1.45^a^	18.50 ± 3.47*
6 Week	11.20 ± 2.62^b^	18.50 ± 3.47*
8 Week	16.60 ± 3.44^c^	18.50 ± 3.47
10 Week	15.50 ± 3.87	18.50 ± 3.47
12 Week	14.20 ± 4.05	18.50 ± 3.47*

### 3.4 Immunofluorescence analysis of type I collagen expression

**FIGURE 5:**
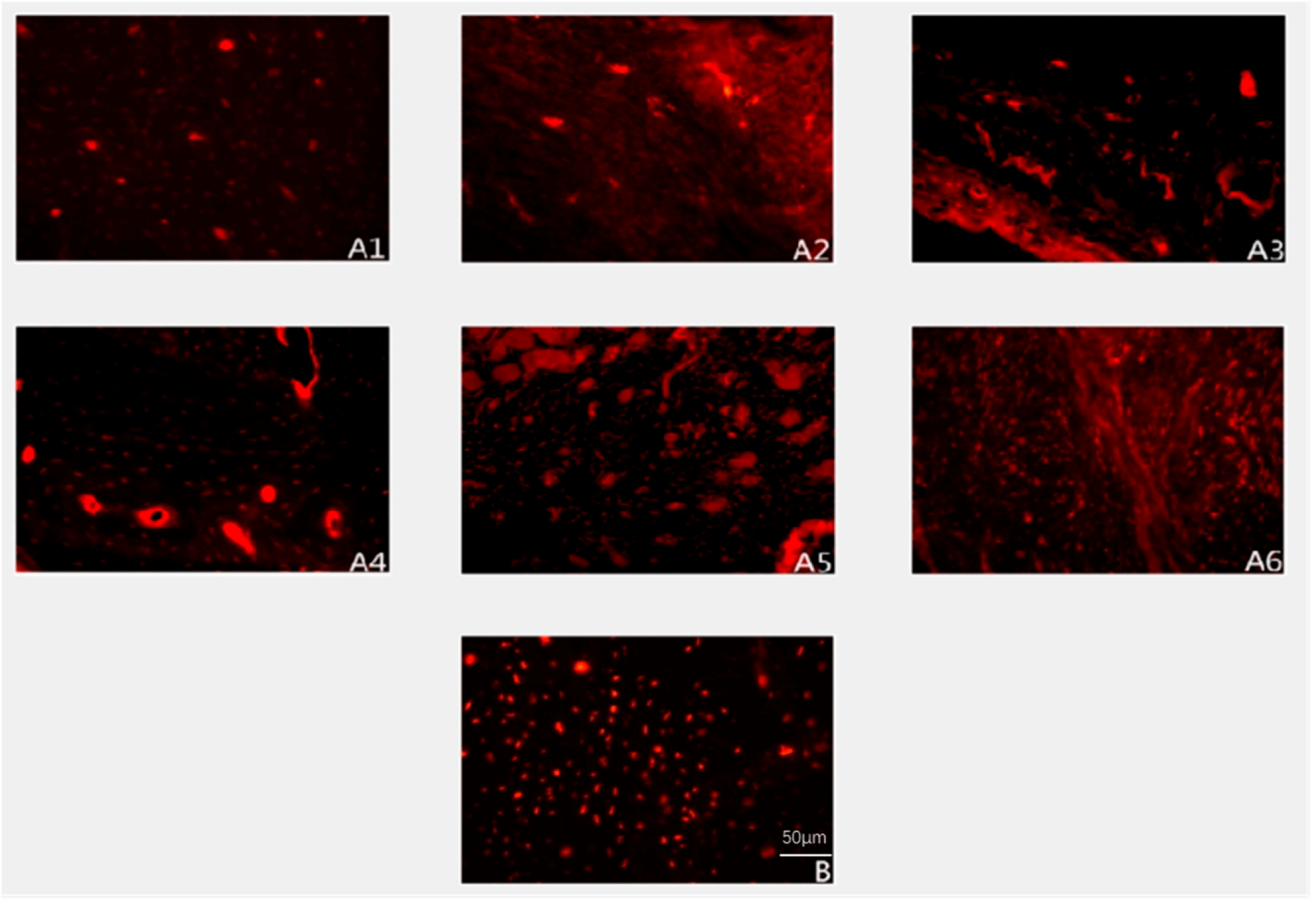
Expression of Type I collagen deposition (immunofluorescence staining ×200). Note: **(A1–A6)** represent 2, 4, 6, 8, 10, and 12 weeks postoperatively in the experimental group; B represents the control group.

The experimental group displayed a biphasic expression pattern of type I collagen immunofluorescence intensity, reaching its peak at week 10 (Figure 5). Within-group temporal analysis revealed progressive and statistically significant increases between all consecutive time points from week 2 to week 10 (week 2 vs. 4: P < 0.05; week 4 vs. 6: P < 0.05; week 6 vs. 8: P < 0.05; week 8 vs. 10: P < 0.05). However, no significant difference was observed between week 10 and 12 (P > 0.05), indicating stabilization of collagen synthesis following the peak phase.

**FIGURE 6:**
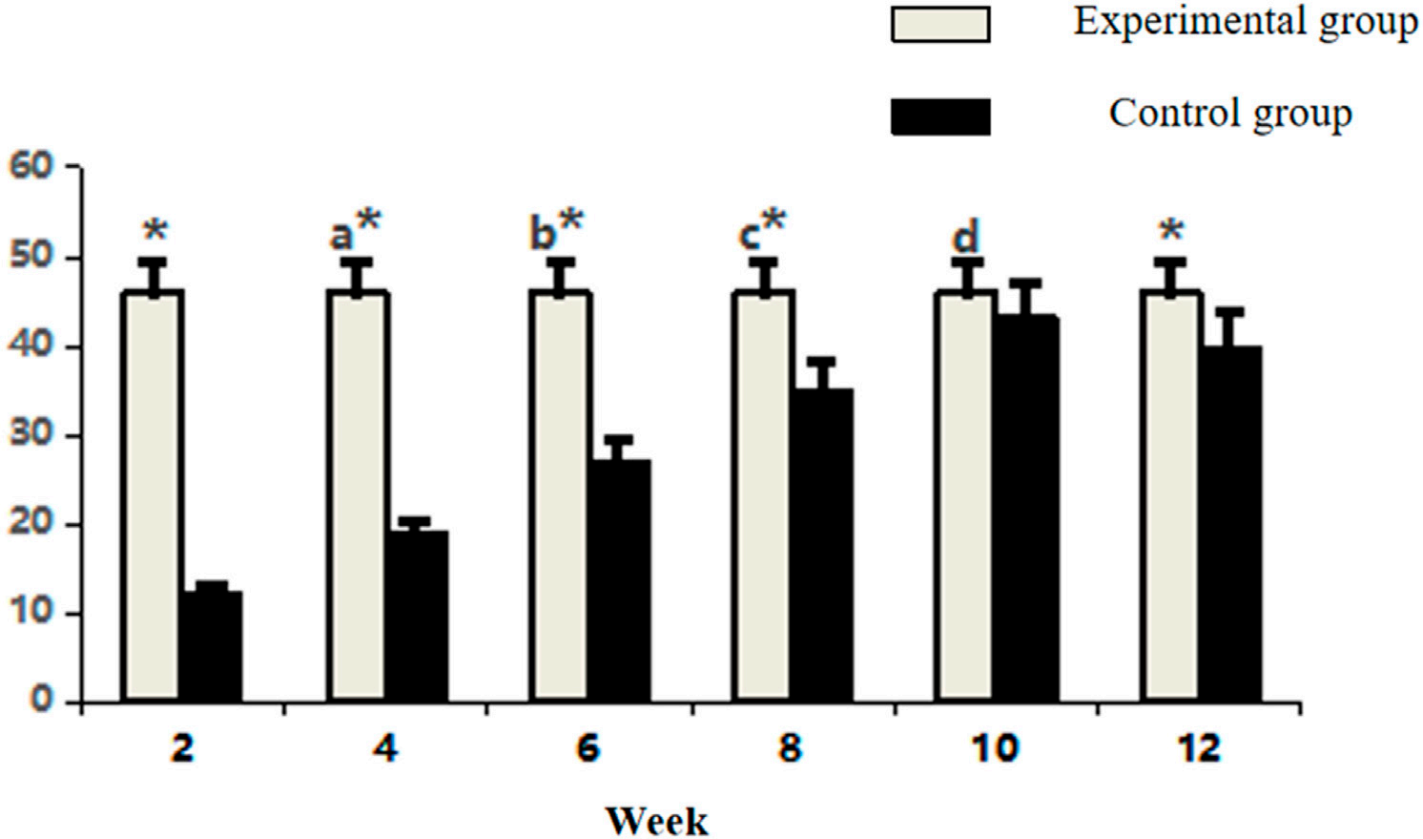
Comparative analysis of Type I collagen fluorescence intensity in osseous tissue. Note: a: 4-week vs. 2-week in Experimental group, P < 0.05; b: 6-week vs. 4-week within group, P < 0.05; c: 8-week vs. 6-week within group, P < 0.05; d: 10-week vs. 8-week within group, P < 0.05; *: Experimental vs. Control group comparison, P < 0.05.

Between-group comparisons demonstrated consistently higher collagen expression in control specimens at early and late stages (week 2, 4, 6, 8, and 12: P < 0.05). Notably, the experimental group achieved transient parity with controls at week 10 (P > 0.05), coinciding with its expression peak (Figure 6; Table 2). This temporal convergence suggests that the prefabrication process temporarily attains native-like collagen deposition kinetics during the critical remodeling phase, followed by a decline to sub-physiological levels.

**TABLE 2:** Comparative analysis of Type I collagen fluorescence intensity in osseous tissue (n = 10,‾x ± s).

Time	Experimental group	Control group
2 Week	12.16 ± 1.06	45.89 ± 5.10^*^
4 Week	18.92 ± 4.33^a^	45.89 ± 5.10^*^
6 Week	26.92 ± 3.52^b^	45.89 ± 5.10^*^
8 Week	34.73 ± 8.41^c^	45.89 ± 5.10^*^
10 Week	43.07 ± 6.02^d^	45.89 ± 5.10
12 Week	39.76 ± 5.81	45.89 ± 5.10^*^

### 3.5 Expression levels of VEGF and BMP-2 in rectus femoris muscle

**FIGURE 7:**
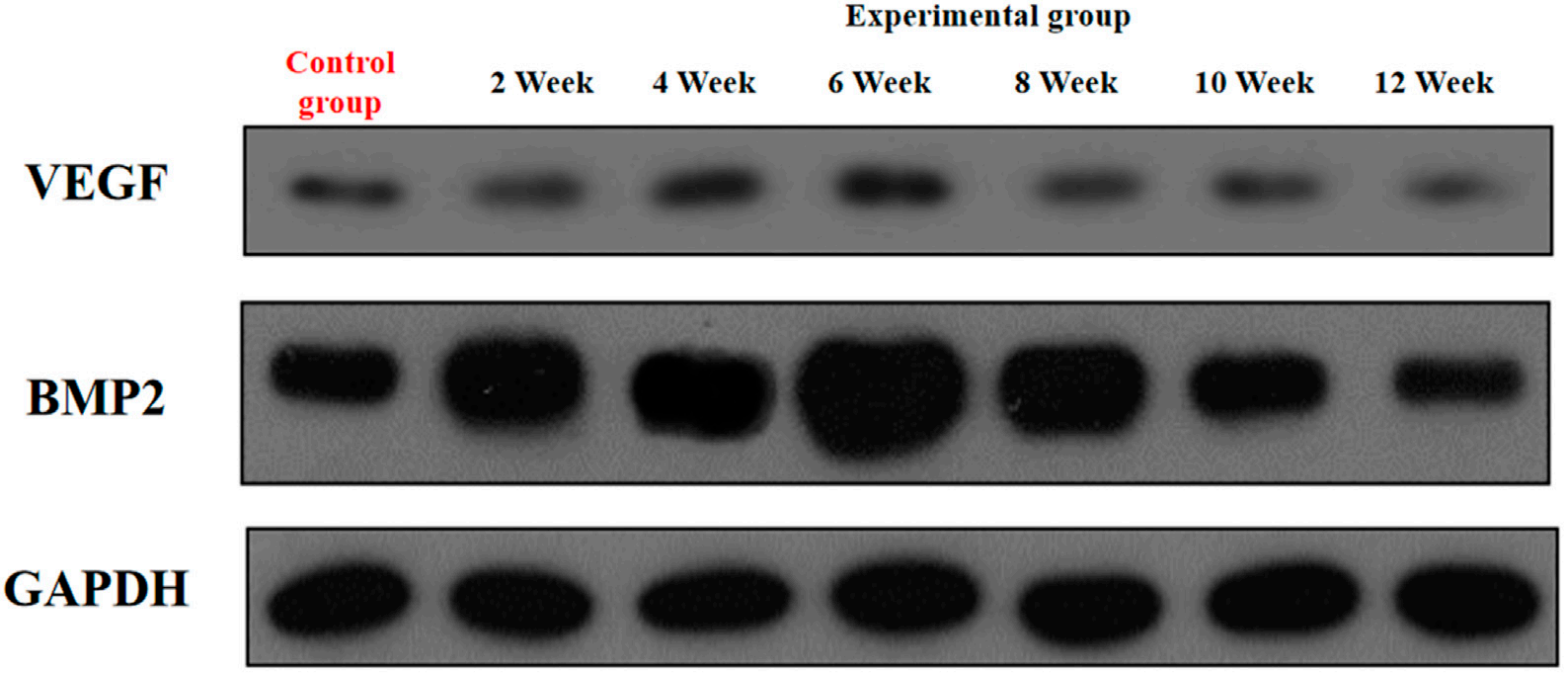
Expression levels of VEGF and BMP-2 secretion in the rectus femoris muscle.

**FIGURE 8:**
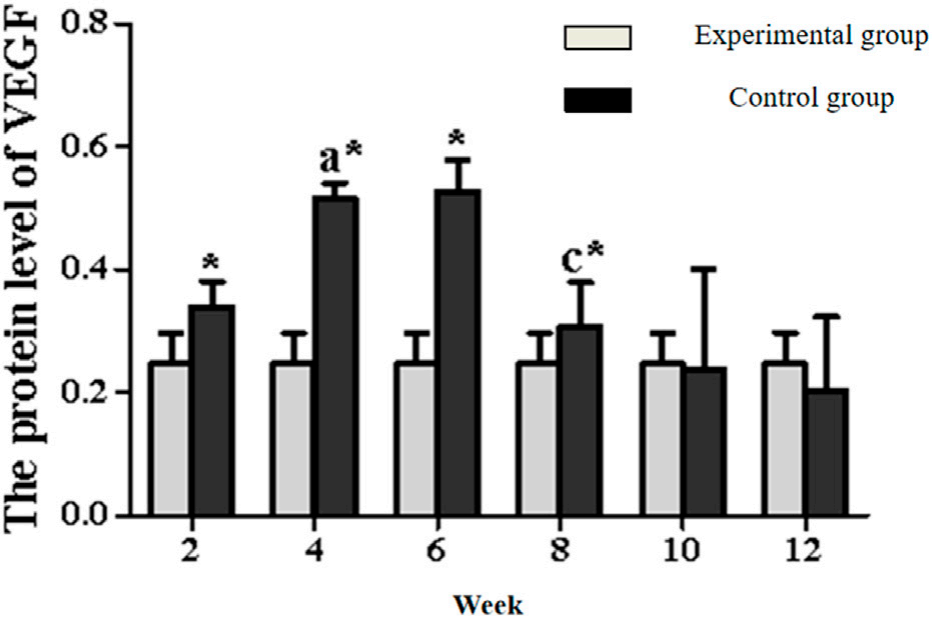
Expression levels of VEGF secretion in the rectus femoris muscle. Note: a: 4-week vs. 2-week in the experimental group, P < 0.05; b: 6-week vs. 4-week in the experimental group, P < 0.05; c: 8-week vs. 6-week in the experimental group, P < 0.05; d: 10-week vs. 8-week in the experimental group, P < 0.05; e: 12-week vs. 10-week in the experimental group, P < 0.05. *: Experimental vs. control group comparison, P < 0.05.

In the experimental group, VEGF exhibited a characteristic temporal pattern: expression peaked at week 4, remained elevated through week 6, and gradually declined from week 8–12 (Figures 7, 8; Table 3). Within-group comparisons revealed a significant increase from week 2–4 (P < 0.05), followed by a marked decrease between week 6 and 8 (P < 0.05). No significant changes occurred between week 8–10 or 10–12 (P > 0.05). Compared to controls, VEGF expression was significantly higher in the experimental group at weeks 2, 4, 6, and 8 (P < 0.05), but converged to control levels by weeks 10 and 12 (P > 0.05).

**TABLE 3:** VEGF/GAPDH (*n* = 10,‾x ± s).

Time	Experimental group	Control group
2 Week	0.340 ± 0.041	0.247 ± 0.050^*^
4 Week	0.516 ± 0.025^a^	0.247 ± 0.050^*^
6 Week	0.527 ± 0.051	0.247 ± 0.050^*^
8 Week	0.307 ± 0.072^c^	0.247 ± 0.050^*^
10 Week	0.238 ± 0.162	0.247 ± 0.050
12 Week	0.204 ± 0.119	0.247 ± 0.050

**FIGURE 9:**
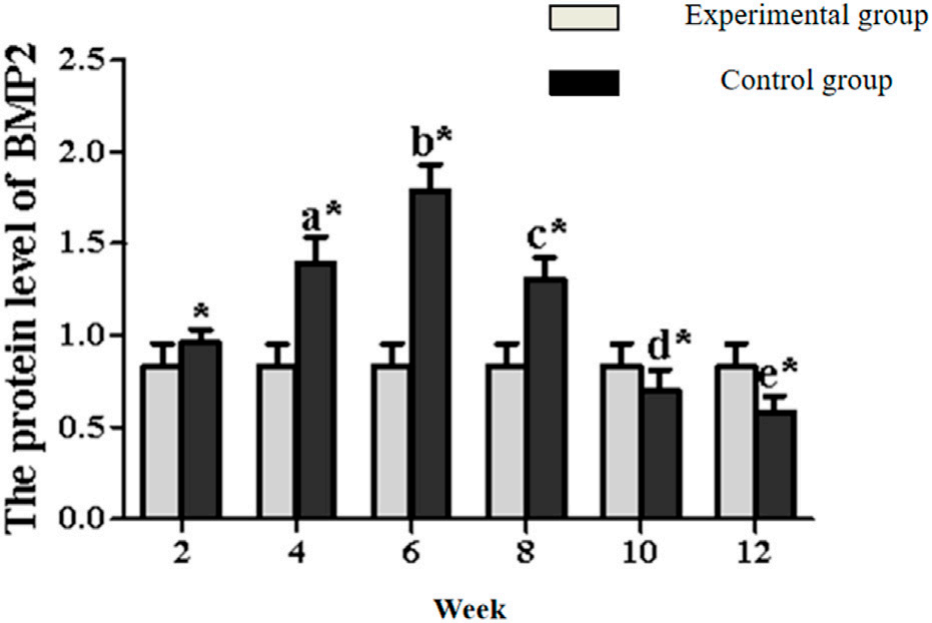
Expression levels of BMP2 secretion in the rectus femoris muscle. Note: a: 4-week vs. 2-week in the experimental group, P < 0.05; b: 6-week vs. 4-week in the experimental group, P < 0.05; c: 8-week vs. 6-week in the experimental group, P < 0.05; d: 10-week vs. 8-week in the experimental group, P < 0.05; e: 12-week vs. 10-week in the experimental group, P < 0.05. *: Experimental vs. control group comparison, P < 0.05.

BMP-2 expression in the experimental group displayed distinct kinetics: levels progressively increased to a peak at week 6, then steadily declined from week 8 onward (Figures 7, 9; Table 4). Significant within-group increments were observed between consecutive time points from week 2–6 (week 2 vs. 4: P < 0.05; week 4 vs. 6: P < 0.05), followed by consistent declines from week 6–12 (all P < 0.05). Between-group analysis showed significantly higher BMP-2 expression in the experimental group at weeks 2–8 (P < 0.05), but lower levels than controls at weeks 10 and 12 (P < 0.05).

**TABLE 4:** BMP2/GAPDH (n = 10,‾x ± s).

Time	Experimental group	Control group
2 weeks	0.960 ± 0.073	0.831 ± 0.124^*^
4 weeks	1.393 ± 0.144^a^	0.831 ± 0.124^*^
6 weeks	1.791 ± 0.141^b^	0.831 ± 0.124^*^
8 weeks	1.305 ± 0.120^c^	0.831 ± 0.124^*^
10 weeks	0.704 ± 0.108^d^	0.831 ± 0.124^*^
12 weeks	0.584 ± 0.086	0.831 ± 0.124^*^

## 4 Discussion

This study systematically elucidates the dual regulatory mechanisms by which skeletal muscle orchestrates angiogenesis and osteogenesis during the ectopic prefabrication of vascularized osteocutaneous flaps. By establishing a novel rabbit model of large autologous bone–muscle composite prefabrication, we demonstrate that spatiotemporal coordination of muscle-derived VEGF and BMP-2 serves as the cornerstone of vascularized bone regeneration. Three key findings emerge: (1) VEGF secretion peaks early (around Week 4–6) in the prefabrication process, driving endothelial migration and neovascularization; (2) BMP-2 secretion peaks slightly later (around Week 6), sustaining osteogenic differentiation and new bone formation; and (3) the progression of vascularization can be delineated into distinct phases—initial ischemia, robust neovascularization by 8 weeks, and a remodeling phase thereafter—which coincide with stages of osteogenesis. These observations extend the “angiogenesis–osteogenesis coupling” theory proposed by Rouwkema et al. (2009). By emphasizing the muscle niche’s dominant role in synchronizing these processes—a paradigm shift from traditional bone-centric regeneration models. Our findings align with emerging perspectives that muscle and bone communicate not only mechanically but also via secretory crosstalk and progenitor cell exchange (Sui et al., 2024). In particular, the temporal decoupling we observed (VEGF preceding BMP-2 by a few weeks) mirrors the natural fracture healing cascade, suggesting that the muscle-mediated prefabrication environment recapitulates critical aspects of physiological bone repair.

The selection of the rectus femoris muscle as a prefabrication carrier proved strategic. This muscle offers a rich population of mesenchymal stem cells (MSCs) (Hu, 2016) and a three-dimensional, well-vascularized architecture (Xiao M. M., 2016) that supports cell survival and fosters dynamic cytokine signaling. Notably, the VEGF–BMP-2 sequence in our model (VEGF peak at 4–6 weeks, BMP-2 peak at 6 weeks) underscores a coordinated angiogenic–osteogenic response. Early VEGF creates an enriched vascular network, which is then capitalized upon by BMP-2-driven osteogenesis—a timing that ensures sufficient blood supply for new bone formation. This muscle-driven temporal hierarchy provides practical insight: clinically, it implies that the optimal window for transplanting the prefabricated flap might be during the proliferative phase (∼4–8 weeks), when angiogenesis is maximal but before BMP-2 and osteogenic activity begin to decline. Transplantation during this window could maximize graft revascularization and osteointegration at the recipient site, taking advantage of peak muscle-derived growth factor activity.

Compared to conventional techniques for large bone defect reconstruction, our approach addresses two persistent challenges: donor site morbidity and delayed “creeping substitution” (slow graft revascularization). By repurposing the patient’s own devitalized bone *in situ* within muscle (rather than harvesting a fresh vascularized graft), we eliminate the need for secondary donor bone harvest and thereby reduce donor site complications. Additionally, by pre-establishing a microvascular network within the graft before final transplantation, we greatly shorten the post-transfer ischemic period. This is evidenced by the relatively rapid establishment of blood supply (by ∼8 weeks in our model, versus 6–12 months in traditional bone grafts without prefabrication). Early revascularization likely also accelerated osteogenic activation, as reflected by earlier ALP/OCN expression in prefabricated grafts in analogous studies (not measured here, but suggested by the early osteocyte appearance). Leonhardt et al. (2009) demonstrated a similar principle in the mandible, where a prefabricated vascularized bone graft successfully restored mandibular continuity after radiation injury, reinforcing the translational potential of muscle–bone composite strategies. For complex infected defects, combining thorough debridement with a customized prefabricated flap could achieve one-stage anatomical and functional restoration—an advance over the current multistage protocols. This concept is echoed by recent regenerative medicine analyses, which highlight the synergy between scaffold-guided bone reconstruction and surgical vascularization strategies (Sparks et al., 2020). Moreover, contemporary reviews emphasize integrating tissue engineering with flap prefabrication to address large defects (Xu et al., 2022), supporting the relevance of our muscle-driven prefabrication approach.

Despite these advances, limitations warrant consideration. First, our rabbit model unavoidably simplifies the complex inflammatory and microbiological milieu of clinical infected bone defects. Second, while VEGF/BMP-2 coordination appears pivotal in this model, the contribution of other muscle-derived factors (e.g., PDGF, FGF-2, TGF-β) remains to be characterized. Third, the relatively short-term follow-up (12 weeks) precludes assessment of long-term outcomes such as ultimate biomechanical strength and the durability of the regenerated bone’s remodeling; these aspects will require further investigation. Fourth, our analyses did not include specialized assessments like Masson’s trichrome staining (for collagen/matrix organization), micro–computed tomography (for three-dimensional bone microarchitecture), or mechanical testing of the regenerated bone segment. While incorporating these techniques could provide additional quantitative validation of bone quality and strength, their absence in this study does not compromise our conclusions. The combination of histological, immunofluorescence, and Western blot data sufficiently demonstrated robust angiogenesis and osteogenesis, and the functional vascularization achieved by 8 weeks strongly implies structural integrity.

Future studies should prioritize: (1) genetically engineered models or inhibitors to dissect the precise signaling pathways of VEGF and BMP-2 (and other myokines) in this context; (2) development of “smart” bioreactor or scaffold systems to modulate cytokine release in real time during prefabrication, potentially enhancing outcomes; and (3) ultimately, clinical trials comparing muscle-prefabricated bone flaps against standard techniques in complex defect reconstruction, to formally evaluate improvements in healing time, graft integration, and patient recovery. Additionally, extending our work, it would be valuable to integrate micro-CT imaging and biomechanical testing in future animal studies to quantitatively correlate vascular and osteogenic parameters with bone strength, and to use Masson’s staining or second-harmonic generation imaging to visualize collagen maturity in the neobone.

In summary, this study provides proof-of-concept that skeletal muscle can serve as an “endogenous bioreactor” to revitalize a devitalized bone segment by sequentially delivering angiogenic and osteogenic stimuli. Our findings contribute to the evolving paradigm of bone–muscle integration in regenerative engineering (Sui et al., 2024), wherein muscle is not merely a passive scaffold but an active driver of bone healing. By demonstrating substantial vascularization and new bone formation within 8–10 weeks (far sooner than traditional grafts) without additional exogenous factors, we highlight a cost-effective and biologically elegant strategy for reconstructing large bone defects. This muscle–bone prefabrication approach, if translated successfully, could transform the treatment of chronic osteomyelitis, segmental bone loss, and other challenging orthopedic conditions by providing personalized, vascularized bone grafts with minimal donor morbidity.

## Data Availability

The original contributions presented in the study are included in the article/supplementary material, further inquiries can be directed to the corresponding author.
